# Framework Nucleic Acids‐Based VEGF Signaling Activating System for Angiogenesis: A Dual Stimulation Strategy

**DOI:** 10.1002/advs.202308701

**Published:** 2024-03-09

**Authors:** Yichen Ge, Qingxuan Wang, Yangxue Yao, Qin Xin, Jiafei Sun, Wen Chen, Yunfeng Lin, Xiaoxiao Cai

**Affiliations:** ^1^ State Key Laboratory of Oral Diseases National Center for Stomatology National Clinical Research Center for Oral Diseases West China Hospital of Stomatology Sichuan University Chengdu Sichuan 610041 P. R. China; ^2^ Sichuan Provincial Engineering Research Center of Oral Biomaterials Chengdu Sichuan 610041 P. R. China

**Keywords:** angiogenesis, aptamer, chemical hypoxia, dimethyloxallyl glycine (DMOG), DNA template click reaction, tetrahedral framework nucleic acids (tFNAs), vascular endothelial growth factor (VEGF)

## Abstract

Angiogenesis is crucial for tissue engineering, wound healing, and regenerative medicine. Nanomaterials constructed based on specific goals can be employed to activate endogenous growth factor‐related signaling. In this study, based on the conventional single‐stranded DNA self‐assembly into tetrahedral framework nucleic acids (tFNAs), the Apt02 nucleic acid aptamer and dimethyloxallyl glycine (DMOG) small molecule are integrated into a complex via a template‐based click chemistry reaction and toehold‐mediated strand displacement reaction. Thus, being able to simulate the VEGF (vascular endothelial growth factor) function and stabilize HIF (hypoxia‐inducible factor), a functional whole is constructed and applied to angiogenesis. Cellular studies demonstrate that the tFNAs‐Apt02 complex (TAC) has a conspicuous affinity to human umbilical vein endothelial cells (HUVECs). Further incubation with DMOG yields the tFNAs‐Apt02‐DMOG complex (TACD), which promotes VEGF secretion, in vitro blood vessel formation, sprouting, and migration of HUVECs. Additionally, TACD enhances angiogenesis by upregulating the VEGF/VEGFR and HIF signaling pathways. Moreover, in a diabetic mouse skin defect repair process, TACD increases blood vessel formation and collagen deposition, therefore accelerating wound healing. The novel strategy simulating VEGF and stabilizing HIF promotes blood‐vessel formation in vivo and in vitro and has the potential for broad applications in the vascularization field.

## Graphical abstract

Angiogenesis is crucial for tissue engineering, wound healing, and regenerative medicine. Here, a framework nucleic acid‐based nanomaterial is synthesized by combining tFNAs, DNA aptamer Apt02, and DMOG to enhance angiogenesis through upregulating the VEGF/VEGFR and HIF signaling in vitro and in vivo.

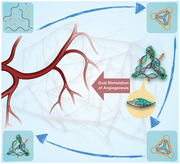

## 1 Introduction

Vascularization plays an important role in tissue engineering, wound healing, and regenerative medicine. Tissue engineering primarily involves developing a 3D composite of cells and biomaterials; thus, the morphology, structure, and function of damaged tissues can be reconstructed to realize permanent replacement, repair injuries, and restore functions.^[^ ^1^, ^2^ ^]^ Reportedly, cells cannot be sustained only through the diffusion of tissue fluid when their distance from a blood vessel is >150–200 µm.^[^ ^3^ ^]^ Oxygen and nutrient supplies must be ensured through the regeneration of blood vessels. Scaffolds, seed cells, and cytokines are fundamental elements in tissue engineering. Owing to the lack of vascular networks in the materials used for tissue engineering scaffolds, the regeneration of blood vessels is a critical process that contributes to forming functional tissue. Therefore, in the development of wound repair as well as tissue regeneration, the primary goal is to prevent ischemic necrosis or poor healing in the central region by promoting vascularization. Growth factors play an indispensable role in vascularization. However, the direct application of exogenous growth factors has certain drawbacks, such as poor circulation, unstable effects, pleiotropic effects, and systemic toxicity, which limit their therapeutic application.^[^ ^4^ ^]^ By employing stimulatory approaches, endogenous vascular‐forming signals within tissues and cells can be continuously activated. This approach could provide a promising strategy for promoting vascularization.

Owing to the aforementioned considerations, this study noted on a novel DNA aptamer (Apt02) and dimethyloxallyl glycine (DMOG). Apt02 is a DNA aptamer isolated by the systematic evolution of ligands via exponential enrichment (SELEX). Reportedly, it binds to VEGFR‐1 and ‐2, thus potentially mimicking the activity of VEGF‐A.^[^ ^5^ ^]^ DMOG, a pro‐angiogenic small molecular drug, competitively inhibits the rate‐limiting enzyme, prolyl hydroxylase (PHD), in the degradation process of the hypoxia‐inducible factor‐1α (HIF‐1α), thereby stabilizing the HIF‐1α under normoxia. Consequently, the effect results in a so‐called chemical hypoxia, which may induce biological effects such as the abundant secretion of VEGF.^[^ ^6^, ^7^, ^8^ ^]^ However, in the application of vascularization, limitations of DMOG such as poor circulation stability, non‐targeting, and short duration of small‐molecule drugs hinder its applications. Therefore, a strategy, aiming to effectively integrate the biological functions of Apt02 and DMOG while overcoming barriers encountered in solo usage, should be developed.

The classic tetrahedral framework nucleic acids (tFNAs) are achieved by self‐assembly of four specific DNA sequences through an annealing process.^[^ ^9^ ^]^ Numerous previous studies have demonstrated the superior properties of tFNAs for cellular proliferation and migration.^[^ ^10^, ^11^, ^12^ ^]^ The specific tetrahedral shape has four apices, which can interact with the cell membrane to trigger “horn attacks”, and ultimately achieve ”self‐reliant“ endocytosis through the caveolin‐mediated pathway.^[^ ^13^ ^]^ Because of its nature of DNA, tFNAs are easy to edit. Previously, several studies have utilized sticky ends to connect functional nucleic acids, such as miRNAs and siRNAs, to the apices of the tFNAs. Theoretically, a tetrahedral framework nucleic acid can connect 1–4 functional nucleic acids at apices. These functional nucleic acids enter cells through the “carrying” effect of the tFNAs to exert their functions. This method has been widely used and yielded acceptable results in previous studies.^[^ ^14^, ^15^, ^16^ ^]^ However, increasingly complex modifications at the apices render the advantage of tetrahedral apices for cellular uptake as useless, as reported in some studies.^[^ ^13^, ^17^ ^]^ In addition, the connection through sticky ends is not absolutely stable, and requires a certain incubation time and temperature, otherwise, the yield of the target product may be considerably affected, or the target structure may even detach from the sticky end before usage.

Considering the potential drawbacks associated with apex modification and sticky ends, in this study, a method to efficiently and stably connect functional nucleic acids to the non‐apex positions of tFNAs through DNA‐templated click reactions was developed. This method enables performing the biological function of Apt02 while maintaining all apices of the tetrahedron. Herein, three Apt02 aptamers were connected using the three edges of the triangular surface of a tetrahedron to build a functional complex referred to as the tFNA‐Apt02 complex (TAC). Additionally, the small‐molecule drug DMOG was incorporated into the TAC structure (TACD) via groove docking based on the DNA double helix. Furthermore, VEGF simulation and chemical hypoxia as dual stimuli were performed to effectively promote angiogenesis.

## 2 Results and Discussion

### 2.1 Design, Synthesis, and Characterization of TAC

**Figure 1:**
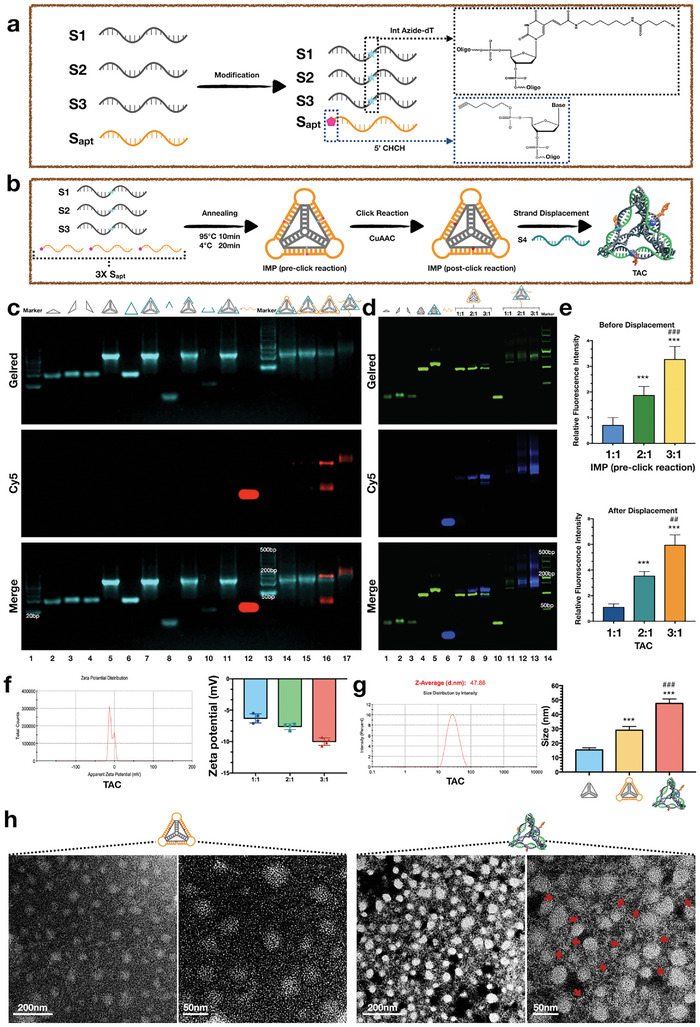
Design, synthesis, and characterization of the TAC. a) Schematic diagram of S1–S3 dT site azide modification and Sapt 5′ end alkyne modification. b) Schematic diagram of the stepwise synthesis of TAC. First, IMP is constructed by S1, S2, S3, and Sapt via a one‐pot annealing process. Subsequently, the click reaction of azide and alkyne catalyzed by Cu(I). Finally, the TAC structure formed through the S4 displacement reaction. c) Agarose gel electrophoresis showing the stepwise construction of the TAC (saturation). The Sapt was successfully modified in the framework nucleic acids as evidenced by the position of Cy5 fluorescence. d) Agarose gel electrophoresis showing the stepwise construction of the TAC (saturation deficit). e) Quantitative analysis of relative fluorescence intensity of IMP (pre‐click reaction) and TAC with different Sapt ratios. Data are presented as the mean ± standard deviation (SD) (n = 3). ^***^ *p* < 0.001 compared with 1:1 group; ^###^ *p* < 0.001, ^##^ *p* < 0.01 compared with 2:1 group (Student's *t*‐test). f) The Zeta potential of TAC decreases as the Sapt ratio increases. g) The size of the basic framework, IMP, and TAC measured using DLS. Data are presented as the mean ± standard deviation (SD) (n = 3). ^***^ *p* < 0.001 compared with basic framework group; ^###^ *p* < 0.001, compared with IMP group (Student's *t*‐test). (h) The TEM image of IMP and TAC. TEM images of TAC reveal branched protrusions surrounding the core structure, being different from IMP.

In TAC nanostructures, the structure consisting of S1, S2, and S3 is referred to as the basic framework; herein, the parts where S1, S2, and S3 pair complementary bases (the three edges extending from one apex of the tetrahedron) are consistent with those in the classic 21 bp tetrahedral framework nucleic acid.^[^ ^11^, ^18^ ^]^ As shown in **Figures** **1**a and S1 (Supporting Information), one dT site is modified by the azide group at each edge, where S1, S2, and S3 do not pair with complementary bases. The edges of the basic framework that do not pair with complementary bases also serve as DNA templates for the click reaction. The 5′ end of Sapt (representing the DNA sequence of Apt02) is modified with an alkyne group. According to Figure 1b, theoretically, in the TAC synthesis process, the basic framework consisting of S1, S2, and S3 was added with Sapt in a 1:3 molar ratio, and an intermediate structure was formed through a one‐pot annealing method; this structure is referred to as an intermediate product (IMP) (pre‐click reaction). In the IMP (pre‐click reaction), the 5′ end of Sapt corresponds to the azide‐modified dT site. Sapt in the IMP forms an incomplete base‐complementary pairing relationship with the basic framework, with 10 bases at the 5′ and 3′ ends of Sapt being complementary to those in the basic framework. After annealing the IMP (pre‐click reaction), the azide‐modified Sapt and alkyne motif undergo a chemical reaction via classical Cu^+^‐catalyzed azide‐alkyne cycloaddition (CuAAC)^[^ ^19^ ^]^ thereby linking Sapt and the basic framework through a chemical bond (IMP, post‐click reaction). For this process, herein, a DNA template‐based click chemistry reaction is utilized. Note that through efficient base pairing of DNA, the distance between the two reactive groups, azide, and alkyne, modified on ssDNA, can be minimized, thereby increasing their effective molar concentration. This enables the click reaction to proceed efficiently at relatively low substrate concentrations.^[^ ^20^, ^21^, ^22^ ^]^ After completing the click reaction, the S4 chain was added, thus triggering a toehold‐mediated DNA strand displacement reaction^[^ ^23^ ^]^ and resulting in the construction of a complete tetrahedral framework. Owing to the previous click chemical linkage, although Sapt was displaced, it could not detach from the tetrahedral framework nucleic acid and essentially formed a functional whole, namely the TAC. Figure 1c,d shows the results of agarose gel electrophoresis, illustrating the stepwise TAC synthesis process and potential outcomes of other chain synthesis methods. In Figure 1c, lanes 1 and 13 represent two different markers. Lanes 2–5 show S1, S2, and S3 and the basic frameworks composed of them. Lanes 6–11 show the results for S4 and various partial S4 bindings within the basic framework. Lane 12 represents Sapt that is labeled with Cy5 fluorescence, which appears red in the electrophoretic images. Lane 14 shows the result of one unit of Sapt with 2/3 of S4 binding to the basic framework; similarly, lane 15 shows the result of two units of Sapt with 1/3 of S4 binding to the basic framework. Evidently, the red fluorescence was weak in this partial S4 and Sapt‐hybridized region. Lane 16 displays the IMP (pre‐click reaction) with an excess amount of partially added Sapt; additionally, note that to occupy all the binding sites of the basic framework with Sapt, the molar ratio of Sapt to the basic framework slightly exceeded 3:1 during the addition. The red strip in lane 16 with faster migration rate was excess‐added unreacted Sapt. The purpose of mildly excessive addition of Sapt was to ensure that the basic framework was fully integrated, thereby increasing the yield of IMP. After annealing, the unreacted Sapt was removed via ultrafiltration; subsequently, complete S4 was added to the remaining IMP (post‐click reaction) to complete the strand displacement reaction, which is shown in lane 17 (TAC). Owing to differences in the 3D structure and molecular weight, the TAC migration rate in electrophoresis was lower than that of the IMP. In Figure 1d, lane 4 represents the tetrahedral nucleic acid framework without Sapt. Lanes 7, 8, and 9 represent the results obtained when Sapt is annealed with the basic framework in molar ratios of 1:1, 2:1, and 3:1, respectively. Lanes 11–13 represent the results of the chain displacement reactions carried out with the aforementioned molar ratios, respectively. Evidently, regardless of whether the basic framework is in a saturated (3:1) or unsaturated state (1:1 and 2:1), tetrahedral framework nucleic acid complexes carrying DNA aptamers could be obtained after the click and chain displacement reactions. After the chain displacement, the migration rate of the product significantly decreases, and the degree of reduction corresponds to the molecular weight of Sapt, without any fluorescent detachment at the position of Sapt. This proves the successful synthesis of TAC. As the Sapt ratio increases, the relative fluorescence intensity of both the IMP and TAC increases (Figure 1e). Dynamic light scattering (DLS) was used to determine zeta potential and particle size. As shown in Figure 1f, with increasing Sapt content, the negative zeta potential of TAC gradually increased. When the ratios of Sapt to the basic framework were 1:1, 2:1, and 3:1, the zeta potentials of TAC were −6.3 ± 0.3651 mV (n = 4), −7.6 ± 0.2415 mV (n = 4), and −10.1 ± 0.2972 mV (n = 4), respectively. The measurement of particle size (Figure 1g) revealed that the size of the hydrated particle in the basic framework was 15.33 ± 0.8819 nm (n = 3), which is close to that of the classical tetrahedral framework nucleic acid. The sizes of the hydrated particles of the IMP and TAC were 29.06 ± 1.528 nm (n = 3) and 47.67 ± 1.764 nm (n = 3), respectively. Transmission electron microscopy (TEM) was used to visually observe the nanoparticle (NPs) morphology (Figure 1h). The IMP is presented as independent particle. In contrast to the IMP, TAC exhibited distinct branched structures. Evidently from the TEM images, the structure of TAC was similar to that of a central core combined with branched protrusions (red arrow). These morphological characteristics align with the anticipated morphology of the framework nucleic acid‐carrying DNA aptamers.

### 2.2 Transportability of TAC and Carrying DMOG of TACD

**Figure 2:**
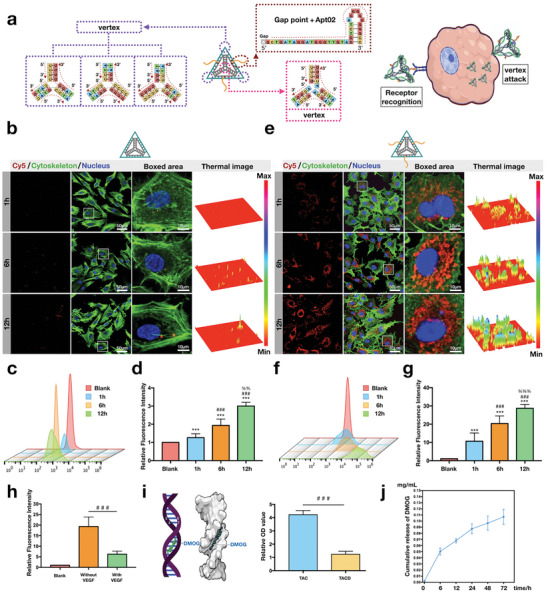
Transportability of TAC and Carrying DMOG of TACD. a) Schematic diagram of four intact apices and three Apt02 aptamers of TAC. b) Fluorescent staining of tetrahedral framework nucleic acids (basic framework connecting with S4 but without Apt02) at 1, 6, and 12 h. c) Flow cytometry and d) Quantitative analysis of relative fluorescence intensity of tetrahedral framework nucleic acids (without Apt02). Data are presented as the mean ± standard deviation (SD) (n = 3). ^***^ *p* < 0.001 compared with the blank group; ^###^ *p* < 0.001 compared with the 1 h group, ^%%^ *p* < 0.01 compared with the 6 h group (Student's *t*‐test). e) Fluorescent staining of TAC at 1, 6, and 12 h. f) Flow cytometry and g) Quantitative analysis of relative fluorescence intensity of TAC. Data are presented as the mean ± standard deviation (SD) (n = 3). ^***^ *p* < 0.001 compared with the blank group; *p* < 0.001 compared with the 1 h group, ^%%%^ *p* < 0.001 compared with the 6 h group (Student's *t*‐test). h) Relative fluorescence intensity of TAC measured by flow cytometry to detect cells cultured with VEGF or without VEGF. Data are presented as the mean ± standard deviation (SD) (n = 3). ^###^ *p* < 0.001 i). Schematic diagram of groove docking and relative OD value of Gel‐Red spectra in the presence of TAC and TACD. Data are presented as the mean ± standard deviation (SD) (n = 3). ^###^ *p* < 0.001 j) The cumulative release of DMOG from TACD. Data are presented as the mean ± standard deviation (SD) (n = 4).

As shown in **Figure** **2**a, TAC preserves the four apices of tetrahedral framework nucleic acid, which serve as the structural basis for its initiation of “horn attack” in endocytosis.^[^ ^24^ ^]^ In the Apt02 sequence, the formation of the G‐quadruplex structure was estimated using the Quadruplex forming G‐Rich Sequences (QGRS) mapper, as reported previously.^[^ ^5^ ^]^ The structure is speculated to be related to the recognition of VEGFR. Next, the interaction between fluorescent materials and cells was compared at different time points, with and without Apt02, using fluorescence staining and flow cytometry to explore the differences induced by structural changes. Figure 2b shows nanoparticles without Apt02 (the product in lane 5 of Figure 1d), where S4 is labeled with Cy5. Evidently, after 1 h of drug treatment, minimal red fluorescence was observed only in few cells. As the drug treatment time was extended to 6 and 12 h, the number of cells exhibiting red fluorescence increased. Similar outcomes were observed by flow cytometry. As the drug treatment time increased, the proportion of cells with Cy5 fluorescence gradually increased (Figure 2c), and the relative fluorescence intensity was also up‐regulated (Figure 2d). This pattern is similar to that reported for classical tFNAs.^[^ ^25^ ^]^ However, the results were different in the presence of Apt02. As shown in Figure 2e, after 1 h of TAC treatment, a more widespread phenomenon of Cy5 fluorescence in conjunction with HUVECs is observed compared with that shown in Figure 2b. The term “in conjunction” is used to describe this phenomenon owing to the possibility that Apt02 binds VEGFR receptors on the cell membrane or it could be an endocytic process. However, for the material itself, both scenarios are expected and considered pathways through which TAC exerts its effects; therefore, we believe that they need not be strictly distinguished. However, based on subsequent experimental results and the time of the endocytosis of tFNAs, the main process was more likely to be the binding of the TAC with VEGFR at 1 h. As the drug treatment time increased to 6 and 12 h, the red fluorescence in the cells became very intense. At these two points, most of the TAC was assumed to be in the cytoplasm, as evident from the distinct boundary observed between the cell nucleus and material through fluorescence staining. This phenomenon typically indicates that the nanomaterial is in the cytoplasm but is unable to enter the cell nucleus. Similarly, flow cytometry analysis revealed that nearly 100% of the cells exhibited red fluorescence after 6 h of Apt02 treatment (Figure 2f). Compared with the blank group, the relative fluorescence intensity of Cy5 increased by 20–30 times after 6–12 h (Figure 2g). The result suggests that compared to the endocytic effect triggered by “horn attacks”, the receptor recognition of cell membrane surface mediated by aptamer can facilitate the binding between nanomaterials and cells. To avoid interference, all growth factors were not added to the culture medium. However, subsequent study aims to determine whether the presence of VEGF affects the binding of TAC with HUVECs. Therefore, cells were cultured beforehand with VEGF and collected for flow cytometry after 1 h. Evidently, the relative fluorescence intensity decreased after the addition of VEGF (Figure 2h). This result could be attributed to the higher affinity of VEGF with VEGFR, compared with that of Apt02. Essentially, in the presence of VEGF, VEGFR preferentially binds to VEGF, which, to some extent, weakens the interaction between TAC and HUVECs. This further suggests that TAC might bind to the VEGFR of cell membrane surface rather than entering the cytoplasm in the early stages. As mentioned, small molecule drugs can be loaded onto DNA nanomaterials through groove docking, adsorption, electrostatic interaction, and hydrophobic forces.^[^ ^10^, ^18^ ^]^ Subsequently, the drug could be released through simple diffusion. DMOG is embedded into the double helix groove region of DNA, similar to the binding of nucleic acid detection molecule GelRed to DNA. TAC and TACD were stained with GelRed, and fluorescence spectra were measured. The fluorescence intensity of TAC was higher than that of TACD, which further confirms the binding between TAC and DMOG. Measurement of the encapsulation efficiency (EE) and loading efficiency (LE) of DMOG in TAC at different mass ratios is shown in Figure S2 (Supporting Information). As the encapsulation efficiency decreased, the loading efficiency increased. Considering the balance between drug loading efficiency and cost, we chose a DMOG concentration of 250 µM, where the EE was ≈57% and the LE was ≈45%. In subsequent experiments, the corresponding ratio (250 nM TAC/250 µM DMOG) was used. At the ratio, the results of in vitro detection of DMOG releasing showed an accumulated volume ≈0.107 ± 0.012 mg mL^−1^ (n = 3) after 72 h.

### 2.3 Influence of TAC and TACD on the Biological Behavior of HUVECs

**Figure 3:**
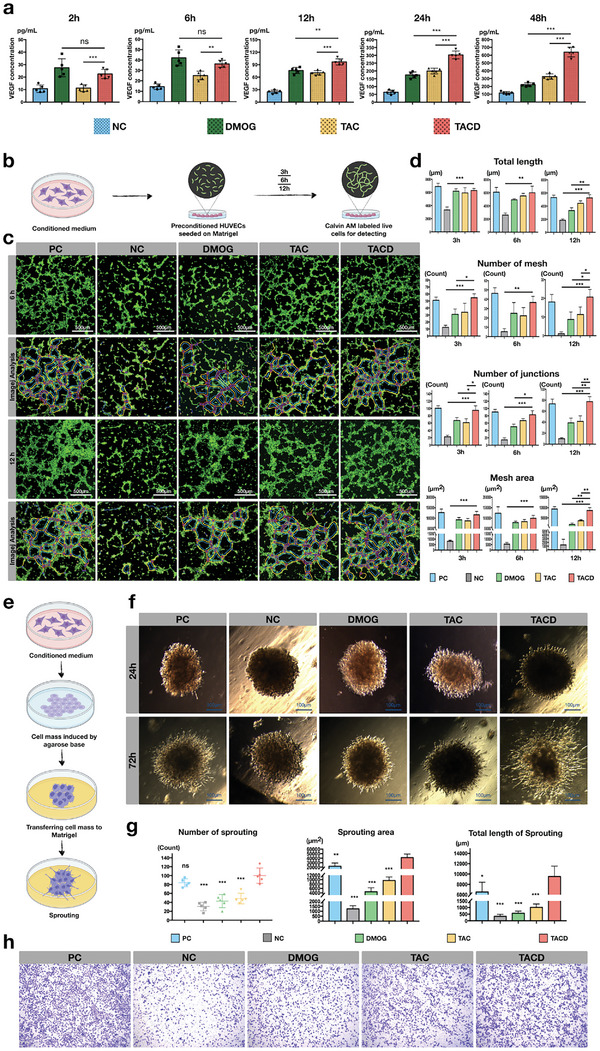
TACD promotes VEGF secretion, tube formation, sprouting, and migration of HUVECs. a) The concentration of VEGF secreted by HUVECs after being cultured with the conditioned medium measured by ELISA. Data are presented as the mean ± standard deviation (SD) (n = 5). ^***^ *p* < 0.001, ^**^ *p* < 0.01(Student's *t*‐test) b) Schematic diagram of tube formation processes. c) The tube network formation of HUVECs and the analytical pictures from ImageJ. d) Quantitative analysis of total length, number of junctions, number of mesh, and mesh area. Data are presented as the mean ± standard deviation (SD) (n = 3). ^***^ *p* < 0.001, ^**^ *p* < 0.01, ^*^ *p* < 0.05 e) Schematic diagram of sprouting processes. f) The sprouting of HUVECs aggregate. g) Quantitative analysis of the number of sprouting, sprouting area, and total length. Data are presented as the mean ± standard deviation (SD) (n = 5). ^***^ *p* < 0.001 compared with TACD (Student's *t*‐test). h) Representative images of the transwell migration assay at 24 h.

Compared with employing exogenous growth factors with unstable effects, promoting the continuous secretion of target cytokines by drug stimulation may be a better strategy for promoting specific biological behaviors.^[^ ^26^, ^27^ ^]^ In this experiment, the concentration of VEGF in the supernatant was detected using an ELISA kit after drug treatment. As shown in **Figures** **3**a, 2h, and 6 h after drug addition, the VEGF level in the small‐molecule DMOG group was slightly higher. However, at 12 h, the VEGF level in the TACD group was higher than that in other groups. At 24 h, the VEGF levels in the TACD group were significantly higher than those in the DMOG and TAC groups. At 48 h, the difference in VEGF levels between the TACD and other groups continued to increase. At this time, the VEGF levels in the negative control, DMOG, TAC, and TACD groups were 115.9 ± 8.112, 222.2 ± 10.55, 326.2 ± 15.39, and 639 ± 27.54 pg mL^−1^, respectively (n = 5). Note that the positive control group contained exogenous VEGF, which was not used in this part of experiment. These results indicate that DMOG as a kind of small molecule drug took a relatively shorter time to embody function after drug administration, and the VEGF level in the DMOG group is higher than other groups before 6 h. These characteristics were consistent with the general characteristics of small molecule drugs, such as being easily able to penetrate cell membranes, working quickly, low specificity, and a short half‐life. Moreover, as administration time increased, the VEGF level of the TAC and TACD groups gradually surpassed the DMOG group. In particular, the most significant increase in VEGF levels in the TACD group was observed after 12 h, which may be related to the sustained effect of TACD‐loaded DMOG. The potential mechanism of TACD involves the sustained release of DMOG and the targeting of VEGFR on HUVECs through Apt02. These effects were significantly slower than the direct reaction of small molecules, but their effects are more remarkable and last longer. Therefore, as time went on, VEGF secretion of TACD group was inverted.

A CCK‐8 effects of the materials and reagents involved in thiseffects of the materials and reagents involved in thiseffects of the materials and reagents involved in this and reagents involved in this experiment on cell viability. In this set of experiments, a buffer group was included to assess whether the buffer used in TAC synthesis has any detrimental effects on cells. As shown in Figure S3 (Supporting Information), the cell viability in the buffer group was not lower than that in the control group at 12, 24, and 48 h. By contrast, the DMOG, TAC, and TACD groups demonstrated varying degrees of positive effects on cell viability. Note that concerns regarding the biological safety of Cu^I^‐mediated CuAAC click reactions have been raised owing to the potentially accumulative toxicity of Cu^I^ in the application of biology.^[^ ^28^ ^]^ However, the DNA‐templated click reaction efficiently increases the effective molar concentration of the reactants.^[^ ^29^ ^]^ This enables the reaction to proceed efficiently at lower substrate and catalyst concentrations (nM level) while avoiding toxic reactions.

The tube formation assay is a commonly used in vitro method for evaluating angiogenesis. In this assay, endothelial cells are cultured on a gel containing reduced growth factor basement membrane extract, and their ability to form 3D capillary‐like structures is measured. During the assay, endothelial cells differentiate and undergo directed migration to arrange, branch, and form tube‐like polygonal networks that reflect the differences in the vasculogenic potential of the stimulating factors.^[^ ^30^ ^]^ The flowchart of the tube formation assay is shown in Figure 3b. Live cell staining images were obtained for tubes formed at 6 and 12 h and analyzed using the ImageJ software to determine the total length, node number, mesh number, and mesh area (Figure 3d). The live cell staining results showed that at 6 h, both the positive control and TACD groups formed dense mesh‐like structures; the TAC group also showed mesh‐like structures but with a relatively lower density. The DMOG group showed more scattered meshes, whereas tube formation was not observed in the negative control group. At 12 h, the mesh density decreased in all groups; however, the trend was consistent with that at 6 h. The ImageJ results show that the TACD group achieved a vasculogenic potential similar to that of the positive control group, whereas that of the TAC group was slightly superior to the DMOG group.

A study on vascular sprouting also employed the method of pre‐culturing cell aggregates under specific conditions for 3D culture (Figure 3e). Cells pre‐cultured under different conditions were induced to form cell aggregates of similar size in agarose gel (Figure S4, Supporting Information). As shown in Figure 3f, the cell aggregates in the TACD group exhibited the most obvious sprouting phenomenon, accompanied by branching structures extending outward. Statistical analysis of the sprouting results using the ImageJ software revealed that the TACD group had the highest number of sprouts, sprouting area, and total sprouting length. This suggests that TACD has a more significant stimulatory effect on sprouting than on tube formation.

Transwell migration assay was conducted to study the chemotactic effect of the nanomaterials on HUVECs (Figure 3h). Evidently, the positive control group with multiple growth factors possessed the highest number of cells that penetrated the polycarbonate membrane. Both the TAC and TACD groups exhibited obvious cell migration. Compared with the negative control group, the number of cells penetrating the membrane was slightly increased in the DMOG group. Analysis of crystal violet staining images using the ImageJ software (Figure S5, Supporting Information) revealed that the number of migrated cells and the stained area in the TACD group were lower than those in the positive control group but significantly better than those in the other groups. Note that the positive control group contains multiple growth factors, such as VEGF, PDGF, and FGF, all of which possess chemotactic effects on HUVECs;^[^ ^31^, ^32^ ^]^ thus, more obvious migration results for the positive control group are reasonable. Based on the results of the DMOG, TAC, and TACD groups, the chemotactic effect of the small molecule DMOG on HUVECs was very limited. Therefore, the VEGF mimic Apt02 is assumed to play a major role in promoting chemotactic migration.

Moreover, the trend of the scratch assay (Figure S6, Supporting Information) was similar to that of the transwell assay, and both TAC and TACD significantly promoted wound healing compared with the negative control. The statistical results from the ImageJ software indicate that the use of DMOG alone as a small molecule does not significantly promote wound healing (Figure S7, Supporting Information). Previously, numerous studies have reported the promoting effects of VEGF on HUVEC proliferation and migration.^[^ ^33^, ^34^ ^]^ Thus, Apt02 carried by TAC and TACD, which mimic the function of VEGF, may activate the downstream signaling pathways of VEGF, potentially explaining the promotion of these biological behaviors. Additionally, numerous previous studies have incorporated DMOG into scaffold materials such as PCL and hydrogels, which instead exhibit a positive effect on migration.^[^ ^35^, ^36^ ^]^ This phenomenon is likely to be related to the sustained release of small‐molecule drugs by the scaffold materials.

### 2.4 TACD Increasing VEGF Signaling and Promoting Angiogenesis Through Two Pathways

**Figure 4:**
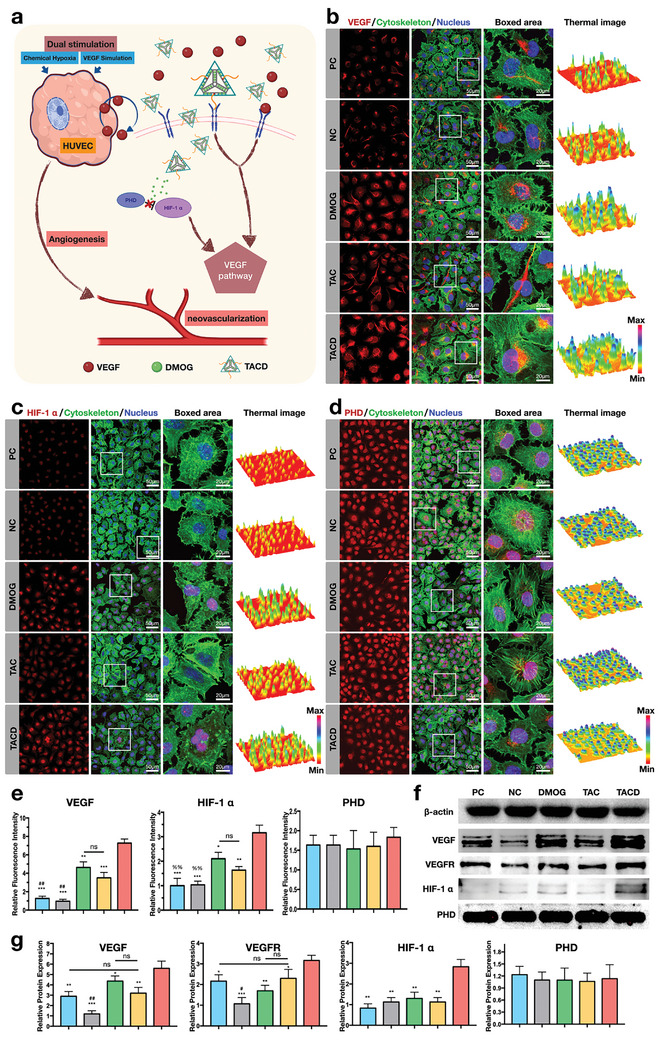
TACD Promoting Angiogenesis Through upregulating VEGF‐A and stabilizing HIF‐1α. a) The Schematic diagram of TACD promoting angiogenesis through dual pathways. b) Immunofluorescence staining of VEGF‐A. The thermal images showing expression level of VEGF‐A. c) Immunofluorescence staining of HIF‐1α. The thermal images showing expression level of HIF‐1α. d) Immunofluorescence staining of PHD. The thermal images showing expression level of PHD. e) Quantitative analysis of fluorescence intensity of VEGF‐A, HIF‐1α, and PHD. Blue, gray, green, yellow, and red represent PC, NC, DMOG, TAC, and TACD, respectively. Data are presented as the mean ± standard deviation (SD) (n = 3). ^***^ *p* < 0.001, ^**^ *p* < 0.01, ^*^ *p* < 0.05 compared with TACD, ^##^ *p* < 0.01, ^#^ *p* < 0.05 compared with TAC, ^%%^ *p* < 0.01 compared with DMOG (Student's *t*‐test). There is no statistically significant difference between groups of PHD. f) Representative western blot images and g) Quantitative analysis of VEGF‐A, VEGFR, HIF‐1α, and PHD. Blue, gray, green, yellow, and red represent PC, NC, DMOG, TAC, and TACD, respectively. Data are presented as the mean ± standard deviation (SD) (n = 3). ^***^ *p* < 0.001, ^**^ *p* < 0.01, ^*^ *p* < 0.05 compared with TACD, ^##^ *p* < 0.01, compared with TAC (Student's *t*‐test). There is no statistically significant difference between groups of PHD.

From the initial design of the nanomaterial, the functions of the DNA aptamer Apt02 and DMOG were integrated with frame nucleic acids. As shown schematically in **Figure** **4**a, the TACD pathway should include both chemically induced hypoxic effects from DMOG and simulated VEGF effects from Apt02. Figure 4b–d depicts the immunofluorescence staining results for VEGF, HIF‐1α, and PHD, respectively. The thermal images obtained through quantitative analysis clearly highlighted the differences in protein expression and distribution characteristics. The results indicate that TCAD significantly promotes the expression of VEGF and HIF‐1α. In particular, HIF‐1α exhibited increased expression in both the nucleus and cytoplasm, whereas the control group exhibited only partial expression in the nucleus and little fluorescence in the cytoplasm. The fluorescence signal for VEGF in the TAC group increased considerably, whereas no significant difference was noted in the fluorescence intensity or distribution of HIF‐1α compared with the control group. Additionally, differences in PHD immunofluorescence staining among the groups were not apparent. The protein relative fluorescence intensity (Figure 4e) revealed that TACD enhanced the expression of VEGF and HIF‐1α, with statistically significant differences compared with other groups. Subsequently, further validation of protein expression was performed using western blot analysis (Figure 4f,g), which demonstrated that the TACD group had the highest VEGF and VEGFR expression levels among the five groups. Additionally, the expression levels of VEGF and VEGFR increased in the DMOG and TAC groups compared with the negative control group. The difference in expression levels of HIF‐1α was also significant, with the TACD group exhibiting the highest expression levels, followed by the DMOG group; by contrast, the expression levels in other groups were low. Similarly, the variation in the expression levels of PHD between the groups was negligible. The concentration of DMOG used in the in vitro experiment was 0.5 mm, and at this concentration, the expression levels of VEGF and HIF‐1α were consistent with the trends obtained when the concentration was used in the former report.^[^ ^37^ ^]^ These results indicate that the effect of TACD is stronger than that of DMOG or TAC alone and that TACD can positively regulate both the VEGF/VEGFR and HIF pathways, both of which promote angiogenesis. Note that PHD is a limiting enzyme for the degradation process of HIF, and the mechanism by which DMOG stabilizes HIF occurs primarily through competitive inhibition.^[^ ^38^ ^]^ Moreover, DMOG does not regulate PHD directly, which may explain why the difference in PHD expression levels among groups was not significant.

### 2.5 TACD Promoting the Healing of Skin Defects in Diabetic Mice

**Figure 5:**
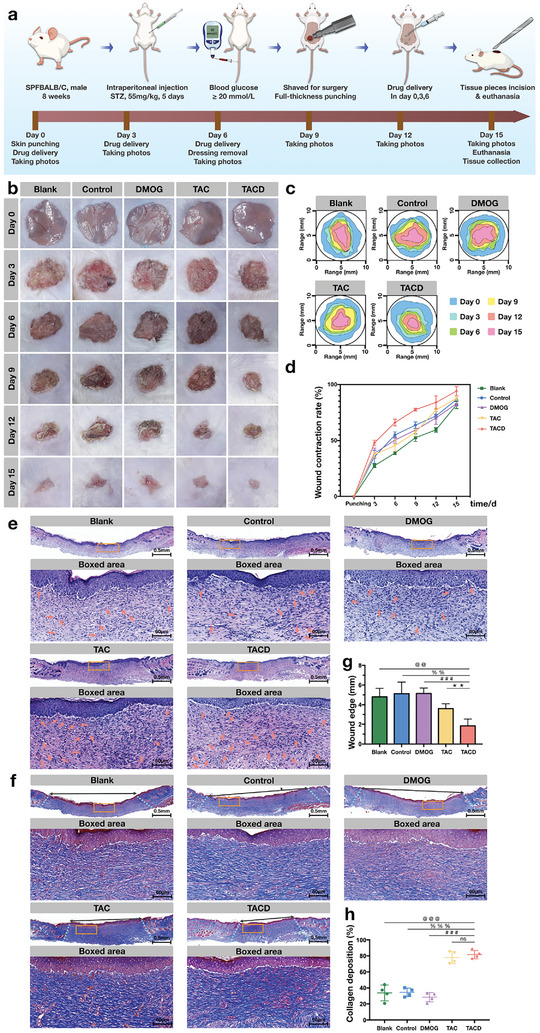
TACD Promoting the Healing of Skin Defects in Diabetic Mice. a) Schematic diagram for the establishment of a full‐thickness skin defect model in diabetic mice and the healing period treatment. b) Representative images of the diabetic wounds at different healing times. c) The merged wound traces of every healing process. d) Trend quantitative analysis of wound contraction during the healing process. Data are presented as the mean ± standard deviation (SD) (n = 3). ^***^ *p* < 0.001 (Student's *t*‐test). e) H&E staining of wound sections in all groups on day 15 f) Masson staining of wound sections in all groups on day 15. g) Quantitative analysis of wound edges. Data are presented as the mean ± standard deviation (SD) (n = 3). ^###^ *p* < 0.001, ^**^, ^@@^, ^%%^ *p* < 0.01, (Student's *t*‐test). h) Collagen deposition on day 15 based on Masson staining. Data are presented as the mean ± standard deviation (SD) (n = 3). ^@@@^, ^%%%^, ^###^ *p* < 0.001, (Student's *t*‐test).

Diabetes‐related vascular disease, also known as diabetic panvascular disease (DPD), is one of the most common diabetic complications.^[^ ^39^ ^]^ DPT is characterized by highly common pathological changes at the vascular level resulting from long‐term hyperglycemic states, with impaired endothelial function being a characteristic feature,^[^ ^40^ ^]^ which has a significant effect on vascular formation. From a vascular perspective, the mechanisms underlying the difficulty in diabetic wound healing compared with normal tissue include continuous hyperglycemia‐induced apoptosis of endothelial cells and poor adaptation to hypoxia (due to high glucose‐induced HIF‐1α destabilization and functional inhibition).^[^ ^41^ ^]^ The mode of action of TACD on vascular endothelial cells combines the positive effects of VEGF on endothelial cells and its stabilizing effects on HIF. Therefore, herein, a full‐thickness skin defect model in diabetic mice was constructed as an in vivo model to investigate the impact of TACD on angiogenesis and whether the relevant in vitro mechanisms also exert effects. **Figure** **5**a displays the experimental procedures conducted at each time point. Herein, hyperglycemia was induced by the continuous injection of STZ. In this experiment, a blood glucose measurement of greater than or equal to 20 mmol L^−1^ was considered as successful model induction and included for subsequent experiments. The drug treatment was administered on the day of model induction, and on the third and sixth days. After 15 days, the mice were euthanized, and samples were collected for further research. To eliminate the potential impact of the buffer on the process of nanomaterial synthesis, the corresponding buffer was applied to the control group. The wound‐healing process is shown in Figure 5b. The skin defect morphology was extracted, and the defect area was calculated using the ImageJ software. The overlap of the defect areas at the six‐time points (Figure 5c) reveals that the TACD group exhibited a significant reduction in defect area in the early stages of the skin defect, thereby achieving the best healing at the time of sample collection. Statistical analysis of the defect area (Figure 5d) revealed that the TACD group achieved a wound healing rate of 47.98 ± 1.299% three days after modeling, whereas the blank, control, DMOG, and TAC groups achieved healing areas of 27.48 ± 0.94%, 35.51 ± 3.37%, 38.67 ± 2.61%, and 37.38 ± 1.33%, respectively. Fifteen days later, the wound contraction rates of the blank, control, DMOG, TAC, and TACD groups were 81.89 ± 1.92%, 86.61 ± 0.80%, 82.9 ± 1.02%, 87.61 ± 0.92%, and 94.18 ± 2.35%, respectively. Slices of the healing area of the skin defect were obtained and stained with HE (Figure 5e) to reveal the boundary between the newly formed skin tissue (with skin appendages) and the original normal tissue; here, the red arrows indicate blood vessels containing red blood cells observed under high magnification. Statistical analysis of the wound edge width (Figure 5g) revealed that at 15 d, the average wound edge of the TACD group was 1.85 ± 0.35 mm, thus indicating better re‐epithelialization. Masson's staining (Figure 5f) and collagen deposition analysis (Figure 5h) further revealed that compared with the other three groups, the TAC and TACD groups exhibited a higher collagen deposition rate.

Evidently from the data, the effect of the control group, in which copper ions were added to the buffer, is acceptable compared with the blank and DMOG groups. We further explored the biosecurity of TAC and TACD through whole‐body administration. After injecting the drugs into the tail vein of mice for seven consecutive days, pathological examinations were performed on the heart, liver, spleen, lungs, and kidneys (Figure S8, Supporting Information). At the histological level, no abnormalities were observed compared to the control group injected with saline. Recent studies demonstrated that certain concentrations of Cu ions can promote autocrine and paracrine effects in HUVECs.^[^ ^42^ ^]^ Moreover, intercellular copper can significantly activate angiogenesis by combining copper transporters with copper chaperones.^[^ ^43^, ^44^ ^]^ Copper ions can bind to endothelial cells and activate the HIF‐1α pathway by simulating a hypoxic environment, regulate the intracellular levels of nitric oxide (NO) and calcium, which are the key factors in vascular tension, and interact with various angiogenic factors such as fibroblast growth factor‐1 (FGF‐1) and heparin.^[^ ^45^, ^46^ ^]^ In this study, owing to the application of DNA template‐based click reactions, the concentration of copper ions was extremely low and may not have demonstrated the aforementioned positive effects. However, note that under the premise of preventing the accumulation of copper ions up to a toxic level, the application of copper ions in angiogenesis should be considered as positive for vascular formation, rather than as potentially toxic.

### 2.6 TACD Accelerating the Healing of Skin Defects in Diabetic Mice by Promoting Angiogenesis

**Figure 6:**
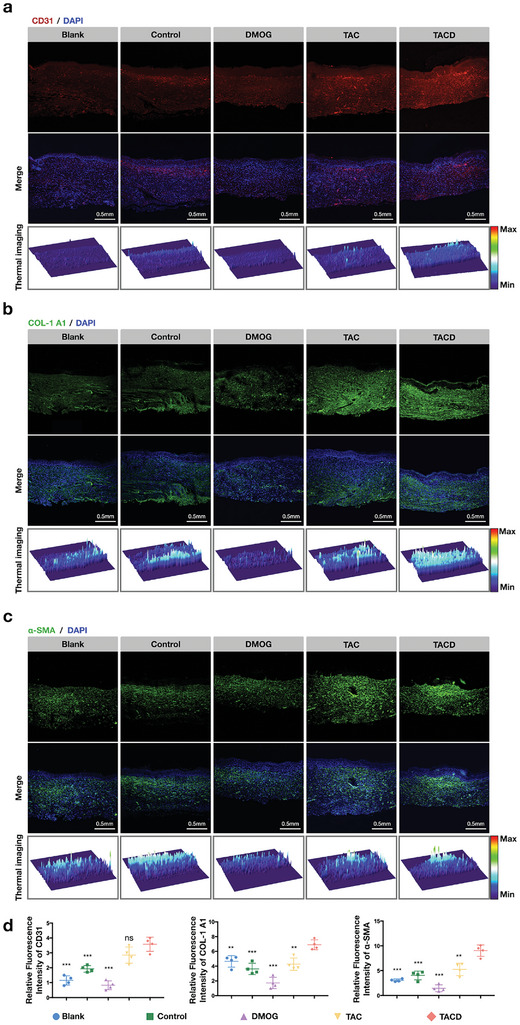
TACD promoting angiogenesis and collagen deposition at the histological level. Immunofluorescence staining a) CD31. b) COL‐1 A1. c) α‐SMA, and d) Quantitative analysis of CD31, COL‐1 A1, and α‐SMA. Data are presented as the mean ± standard deviation (SD) (n = 4). ^***^ *p* < 0.001, ^**^ *p* < 0.01 (Student's *t*‐test). The thermal images showing expression level of the corresponding protein.

Neovascularization is crucial for improving the microenvironment of diabetic wounds, delivering nutrients, and promoting wound healing. After observing the accelerated healing of skin defects in the experimental group macroscopically, angiogenesis was further investigated at the histological level, and the correlation between accelerated skin defect healing and angiogenesis was explored. Immunofluorescent staining for platelet endothelial cell adhesion molecule‐1 (CD31) (**Figure** **6**a), alpha‐smooth muscle actin (α‐SMA) (Figure 6b), and collagen I (COL1A1) (Figure 6c) was performed, and the staining results were quantitatively analyzed using ImageJ (Figure 6d). The TACD group, which carried both Apt02 and DMOG, exhibited significantly higher levels of angiogenic markers compared with the other groups. Additionally, intense CD31 expression was observed in the newly formed skin, particularly in areas undergoing re‐epithelialization, thus indicating the presence of dense vascular tissue in these regions. The TAC group showed scattered areas of high CD31 expression. In the control group, re‐epithelialized areas exhibited partial CD31 expression that was higher than that in the surrounding tissue; however, this phenomenon was not prominent in the blank and DMOG groups.

Additionally, the expression of α‐SMA was increased in the core area of re‐epithelialization and underlying tissue of the TACD group, which may be a key point in promoting wound contraction and healing,^[^ ^47^ ^]^ thus indicating an active process of angiogenesis. Note that during skin defect healing, collagen deposition is directly related to the level of vascularization. The TACD group showed more mature collagen deposition, orderly collagen organization, and higher expression levels of col1a1, representing collagen I, compared with the other groups.

**Figure 7:**
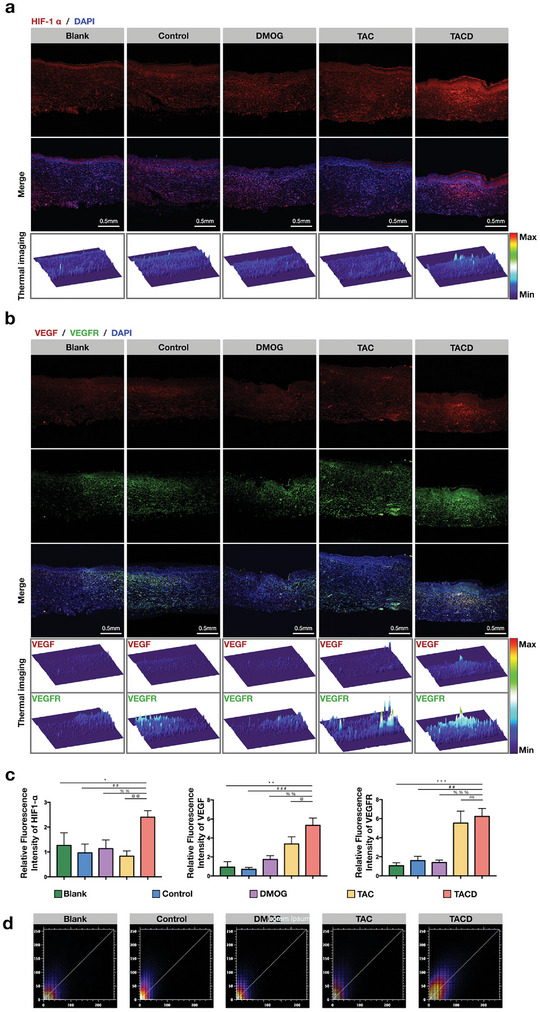
TACD promoting angiogenesis and accelerates wound healing through the upregulation of HIF‐1α and VEGF/VEGFR. Immunofluorescence staining a) HIF‐1α, b) VEGF/VEGFR, and c) Quantitative analysis of HIF‐1α, VEGF‐A, and VEGFR2. Data are presented as the mean ± standard deviation (SD) (n = 4). ^***^, ^###^, ^%%%^, ^@@@^ *p* < 0.001; ^**^, ^##^, ^%%^, ^@@^ *p* < 0.01; ^*^, ^#^, ^%^, ^@^ *p* < 0.05 (Student's *t*‐test). The thermal images showing expression level of the corresponding protein. d) Fluorescence co‐localization of VEGF/VEGFR.

**Figure 8:**
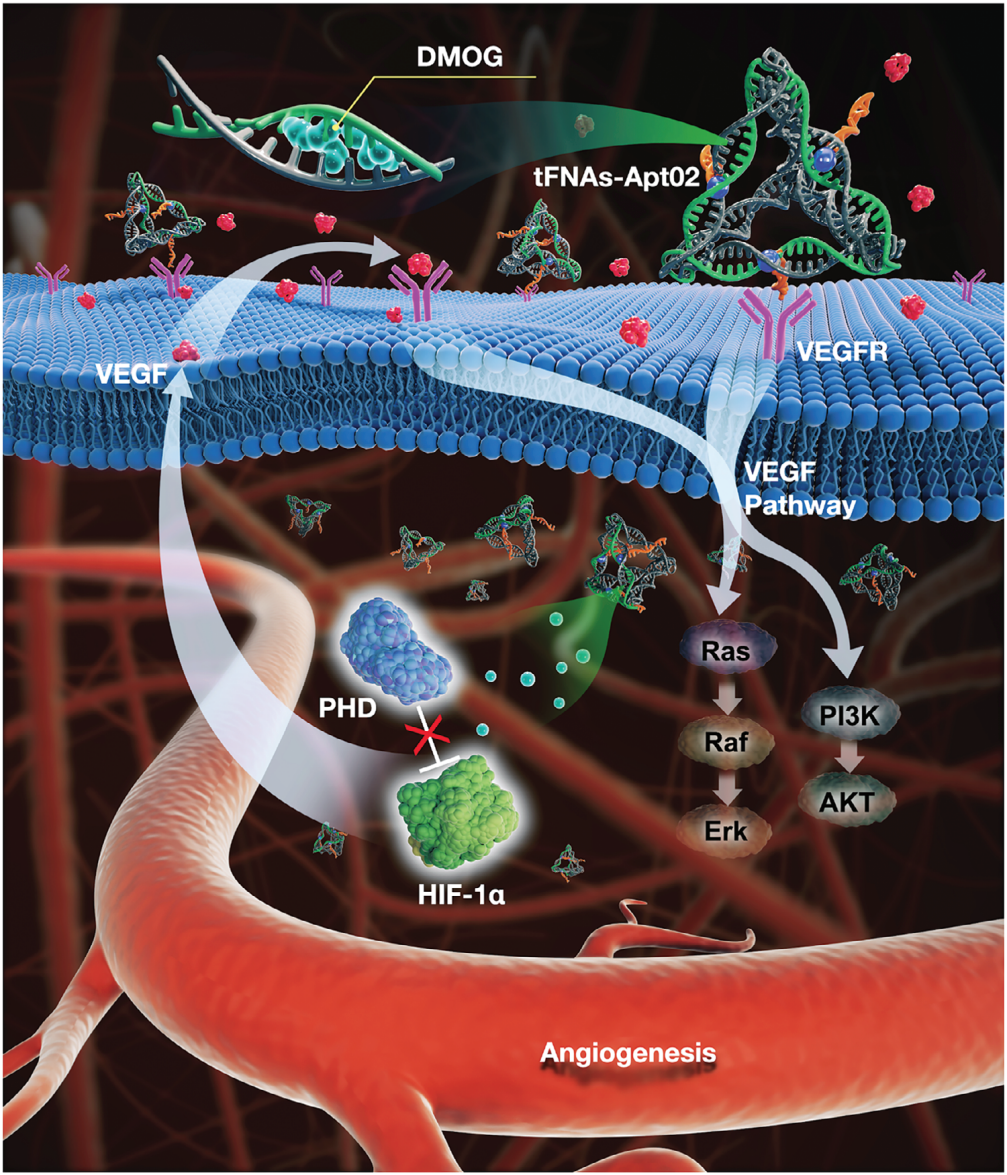
Schematic illustration of composition and structure of TAC and TACD as well as their mechanisms on angiogenesis. Based on the conventional single‐stranded DNA self‐assembly into framework nucleic acids (tFNAs), the Apt02 nucleic acid aptamer and dimethyloxallyl glycine (DMOG) small molecule are integrated into a complex via a template‐based click chemistry reaction and toehold‐mediated strand displacement reaction. TAC is a functional whole that simulates the VEGF function, and stabilized HIF is constructed. Further incubation with DMOG yields TACD, which promotes VEGF secretion by HUVECs, in vitro blood vessel formation, sprouting, and cell migration. Additionally, TACD enhances angiogenesis by upregulating the VEGF/VEGFR and HIF signaling pathways.

Consistent with in vitro experiments, the expression levels of HIF1‐α (**Figure** **7**a), VEGF, and VEGFR (Figure 7b) at the tissue level were tested, and quantitative analysis (Figure 7c) was performed. Evidently, the TACD group showed significantly higher HIF1‐α fluorescence intensity in the supraepithelial and subepithelial regions compared with the other groups. However, the DMOG group, which may be related to HIF1‐α, did not exhibit any differences in fluorescence distribution and intensity compared with the other groups. In the regions where HIF1‐α was highly expressed, the TACD group also showed higher fluorescence intensity of VEGF. Simultaneously, VEGFR showed high expression levels in the newly formed skin. The expression levels of VEGF and VEGFR were significantly higher in the TAC group than those in the other three groups. The correlation between the positions of VEGF and VEGFR was analyzed using the ImageJ software (Figure 7d); evidently, the TACD group showed a higher fluorescence signal overlap with the diagonal, thus indicating that the co‐localization phenomenon of VEGF and VEGFR fluorescence was more pronounced in the TACD group. A possible explanation for this is that the increase in VEGFR expression is more likely due to the increase in VEGF expression, which we believe is directly related to TACD stabilizing HIF and simulating VEGF function (**Figure** **8**).

In the detection of skin defect healing in diabetic mice, the effect of the sole application of DMOG was not as expected. This unexpected result was attributed to the low stability of small molecules in the tissue microenvironment as well as a relatively low concentration of DMOG used in the experiment. The concentration of DMOG used in the in vivo experiments in this study was 0.8 mm (0.14 mg mL^−1^), while the effective concentration in vivo to promote soft tissue healing mentioned in previous research is ≈1 mg mL^−1^.^[^ ^37^ ^]^ We believe that the significant difference in concentration is the main reason for the significant changes of DMOG group in the experiment compared to the former report. However, the cumulative amount of DMOG released from the TACD group at 48 h was less than 0.1 mg mL^−1^, herein the corresponding concentration of DMOG chosen should be at the same level to maintain comparability. Due to the sustained release of DMOG by TACD and its targeting of HUVECs, it has been shown to be more effective than the solo application of DMOG. DMOGs have been integrated into tissue‐engineered scaffold materials to enhance their long‐term performance.^[^ ^48^, ^49^, ^50^ ^]^ DMOG molecules bound to the DNA skeleton of TAC can exert a protective effect by binding to nucleic acid nanostructures. However, this binding is not a strong chemical reaction but rather a weak binding based on DNA grooves, which can act similarly to sustained release.^[^ ^51^ ^]^

## 3 Conclusion

With the development of tissue engineering and regenerative medicine, research concerning promoting vascularization and its mechanisms is continuously increasing. This study focused on integrating the VEGF mimics DNA aptamer Apt02 and HIF1‐α stabilizer DMOG into a functional entity based on a DNA framework nucleic acid structure. Furthermore, considering the limitations of the traditional method for modifying functional nucleic acids through sticky ends, azides and alkynes were introduced into the basic framework and Apt02, respectively. Through template‐based click chemistry and toehold‐mediated strand displacement reactions, the Apt02 nucleic acid aptamer was chemically modified onto the central edge of one triangular surface of the tetrahedral framework nucleic acid skeleton to construct a completely new structure named TAC. Subsequently, taking advantage of the double‐helical structure of DNA frame nucleic acids, the small‐molecule drug DMOG was combined with the groove docking through incubation, thus resulting in the successful synthesis of the TACD structure. Owing to the recognition of VEGFR by the Apt02 nucleic acid aptamer carried by the TAC structure, this structure exhibits considerably high affinity for endothelial cells compared with framework nucleic acids without Apt02. The TACD nanomaterial prepared in this study can promote the secretion of VEGF by HUVECs and improve a series of biological processes including vascular formation, sprouting, and cell migration in vitro. Mechanism studies have shown that TACD enhances vascular formation by upregulating the VEGF/VEGFR and HIF signaling pathways. When applied to a diabetic mouse skin defect model, TACD enhanced angiogenesis, collagen deposition, therefore accelerating wound healing. At the histological level, the expression of VEGF, VEGFR, and HIF was upregulating. In summary, this study proposed a novel strategy for promoting vascularization based on framework nucleic acids to construct a non‐apex modified DNA nano‐structure which included DNA aptemer Apt02 and DMOG. This approach promotes vascular formation both in vivo and in vitro by upregulating VEGF and stabilizing HIF and it also may have broad prospects for application in the field of tissue engineering vascularization.

## 4 Experimental Section

### Assembly and Characterization of TAC and TACD

The raw materials and sequence modification necessary for the synthesis of TAC were purchased from Sangon Biotech (Shanghai, China). All oligonucleotide sequences used in the experiment are presented in the supporting information (Figure S1, Supporting Information). Equal concentrations of S1, S2, S3, and Sapt were added to TM buffer (50 mM MgCl_2_·6H_2_O and 10 mM Tris–HCl, PH = 8.0) in a ratio of 1:1:1:3, respectively. The mixed nucleic acid material was heated to 95 °C and held for 10 min, then rapidly cooled to 4 °C and maintained for 20 min to form IMP (pre‐click reaction). Other framework nucleic acid structures mentioned in the experiment were also made using the annealing program. The CuAAC solution for catalyzing the click reaction was prepared in a volume ratio of 4:1:5 with BTTAA (10 mm), CuSO4 (5 mm), and sodium ascorbate (10 mm). IMP (pre‐click reaction), PBS buffer (0.01 m), and CuAAC solution were mixed in a volume ratio of 8:1:1, shaken, and reacted at 37 °C in a thermal cycler for 6 h to form IMP (post‐click reaction). S4 was added and incubated at 37 °C for 4 h, then followed by ultrafiltration to obtain TAC. The DMOG (HY‐15893‐27218, MCE, USA) solution was added to the TAC solution (250 µm DMOG and 250 nm TAC for cellular application concentration), mixed, and shaken for 6 h at room temperature. The resulting mixture was then ultrafiltrated to obtain TACD. Subsequently, the single‐strand DNA, synthesized TAC, and all intermediate products were analyzed using 3% agarose gel electrophoresis (AGE) (120 V, 30 min). The topography of IMP and TAC was observed using TEM (Tecnai G2 F20 S‐TWIN, FEI). The size and potential of TAC were determined using a Zetasizer Nano ZS90 system (Malvern, United Kingdom).

### Calculation of Encapsulation and Loading Efficiency

Unloaded DMOG molecules were measured through ultrafiltration and the strand curve. The encapsulation efficiency (EE) and the loading efficiency (LE) were calculated as follows:

(1) $$\mathrm{encapsulation}\;\mathrm{efficiency}\;\mathrm{of}\;\mathrm{DMOG}\;\left(\%\right)=\frac{\mathrm{initial}\;\mathrm{mass}\;\mathrm{of}\;\mathrm{DMOG}-\mathrm{residual}\;\mathrm{mass}\;\mathrm{of}\;\mathrm{DMOG}}{\mathrm{initial}\;\mathrm{mass}\;\mathrm{of}\;\mathrm{DMOG}}\times 100\%$$

(2) $$\mathrm{loading}\;\mathrm{efficiency}\;\mathrm{of}\;\mathrm{DMOG}\;\left(\%\right)=\frac{\mathrm{initial}\;\mathrm{mass}\;\mathrm{of}\;\mathrm{DMOG}-\mathrm{residual}\;\mathrm{mass}\;\mathrm{of}\;\mathrm{DMOG}}{\mathrm{total}\;\mathrm{mass}\;\mathrm{of}\;\mathrm{nanoparticles}}\times 100\%$$

### Cultivation and Experimental Stimulations of HUVECs

In the experiment, 6–10 passages of HUVECs were used and cultured in ECM (5% serum, 1% EGCS, 1% Penicillin‐Streptomycin Solution, Sciencell, USA) medium, incubated in an incubator (37 °C, 5% CO2). Prior to drug treatment, starvation treatment was performed using a serum‐free, growth factor‐free medium. The experimental groups included a positive control (PC) with the addition of 8 ng mL^−1^ rHuVEGF165 (Prime Gene, Shanghai), a negative control (NC) using a single serum‐free, growth factor‐free medium, the DMOG group with the addition of 500 µM drug in the medium, and the TAC and TACD groups with the addition of 250 nM nanomaterials in the medium.

### Flow Cytometry

Quantitative analysis of nanomaterials labeled with Cy5 uptake by cells was performed using a flow cytometer (FC500, BECKMAN). After drug incubation (1, 6, and 12 h) was completed, cells were washed with PBS, detached by scraping, dispersed, and filtered. The fluorescence intensity of cells was measured using a flow cytometer to determine the cellular uptake of nano‐materials. Fluorescence analysis was performed using FlowJo software.

### Quantification of DMOG Release

The collected supernatants of every time point after TACD ultrafiltration were detected via spectral detection at a wavelength of 230 nm using a spectrophotometer (NanoPhotometer N60, Implen, Germany), and the value referred to the established DMOG standard curve.

### Detection of VEGF Secretion

The Human VEGF Elisa kit (NOVUS, Bio‐Techne of China) was used, and the reagents were prepared on ice according to the manufacturer's instructions. In brief, different concentrations of standard samples were added to each well to generate a standard curve. The test samples were added to the wells and incubated at 37 °C for 120 min. Then, 100 µL of antibody working solution, 100 µL of enzyme conjugate working solution, and 100 µL of chromogenic substrate were added to each well sequentially. After terminating the reaction, the absorbance of the samples was measured at 450 nm, and the protein concentrations of the sample were calculated.

### Tube Formation Assay

The protocol for the in vitro tube formation assay was based on previous reports.^[^ ^17^ ^]^ Briefly, precooled IF cell dishes were coated with Matrigel (Corning, no. 356234) and incubated at 37 °C until they solidified. Conditioned HUVECs were seeded at a density of 5 × 10^4^ cells per 100 µL. A 150 µL cell‐resuspended medium was added to the dish. At 3, 6, and 12 h, 50 µL of fresh medium with Calcein‐AM (0.2 µL) (abcam, ab141420) was added. After incubation for 5 min, the samples were observed using an inverted fluorescence microscope (Leica, DMi8).

### Vascular Sprouting Assay

A 1.5% agarose solution was prepared and added to each well of a 96‐well plate for 50 µL. The plate was allowed to sit at room temperature for 20 min until the agarose cooled and solidified completely. Cell suspensions were prepared and added 100 µL to the prepared plates (2000 cells). The cell cluster images were captured using a microscope. The melted matrigel (Corning, no. 356234) was spread onto the plate wells and placed in an incubator at 37 °C for 30 min. The formed cell clusters were carefully removed and placed onto the solidified gel surface; the clusters were cultivated, and the sprouting images were captured using a microscope (Leica, DMi8) at 24 and 72 h. The sprouting area, number of sprouting, and length of sprouting were analyzed by using ImageJ software.

### Transwell Migration Assay

HUVECs were seeded in transwell chambers (8 µm, 24‐well plate, Coring), and corresponding drugs were added separately to the lower chamber. The cells were cultured in an incubator for 24 h. After removing the remaining cells in the upper chamber, the substratum of the polycarbonate membrane was stained with crystal violet, and the number of migrated cells was observed and statistically analyzed under an optical microscope (Leica, DMi8).

### Cell Viability Assay

After the cells were adhered to the wall, the experimental groups were treated with drugs and cultured for 12, 24, and 48 h, respectively. The CCK8 working solution (HY‐K0301, MCE) was prepared according to the manufacturer's instructions and added to the wells at a volume of 200 µL per well. The plate was then incubated in the dark for 1 h in a cell culture incubator. The absorbance value was measured at a wavelength of 450 nm.

### Scratch Assay

HUVECs were seeded in 6‐well plates and allowed to grow until cell confluence reached 100%. Subsequently, a straight scratch was made using a 200ul yellow pipette tip, and residual cell debris was washed off with PBS. ECM medium and drug treatment were then added, and cell migration images were captured at 0, 24, and 48 h under a microscope. The degree of wound closure was analyzed using ImageJ software.

### Immunofluorescence (Cytology)

HUVECs with good growth status were inoculated in confocal dishes, processed, and sampled. 4% PFA was fixed for 10 min, followed by PBS wash. 0.25% Triton X‐100 was used for permeabilization for 15 min. After PBS washing, 5% BSA was used for blocking for 1 h. The primary antibody dilution liquid was added and incubated overnight at 4 °C. The primary antibodies used were HIF‐1 alpha (ab51608, Abcam); VEGFA (ab52917, Abcam); PHD (ab113077, Abcam). On the second day, after PBS washing, fluorescence secondary antibodies were added and incubated at room temperature for 2 h. After PBS washing, FITC staining solution was added and incubated overnight at 4 °C. Finally, a DAPI staining solution was added and incubated for 10 min, followed by glycerol sealing. The laser confocal microscope was used for image acquisition, and ImageJ software was used for analysis and statistics.

### Western blot

SDS‐PAGE electrophoresis: The protein sample and loading buffer were mixed in a 1:1 ratio and heated at 100 °C for 5 min to denature the protein. The electrophoresis buffer was prepared, and the sample was loaded. The gel ran at a constant voltage of 60 V for 30 min, then 110 V for 60 min to completely separate the target protein. Transfer and blocking: The PVDF membranes were activated with methanol. The membrane was transferred at a constant current of 250 mA for 90–120 min and then washed for 5 min with TBST before being blocked with 5% skim milk powder for 60 min. The primary antibody was diluted according to the instructions using the appropriate antibody dilution buffer and incubated overnight at 4 °C. Primary antibodies used were: HIF‐1 alpha (ab51608, Abcam); VEGFA (ab46154, Abcam); VEGF Receptor 2 (ab2349, Abcam); PHD (ab113077, Abcam). The secondary antibody was incubated at room temperature for 1 h. Development: The developing solution was added, and the gel was exposed and saved in an imaging system. The protein band density values were analyzed using ImageJ.

### Experiment on the Healing of Skin Defects in Diabetic Mice

All surgical procedures strictly followed guidelines approved by the Sichuan University of Animal Experimental Ethics Committee. (The ethics approval number: WCHSIRB‐D‐2023‐648) This study included 20 male SPFBALB/C mice aged 8 weeks with successful diabetes modeling, randomly divided into five groups. The mice were anesthetized using isoflurane (Lunanbeite Pharmaceutical Co. LTD., China). After shaving the back area, the skin was washed with a povidone‐iodine solution and wiped with alcohol. A circular, full‐thickness skin defect was created on the back of each animal using a circular template with a diameter of 10 mm. A circular ring piece (Ø 13 mm) was used to secure the wound and limit wound contraction in rodents. The blank group did not receive any drug treatment, the control group received synthesized buffer as a control, the DMOG group received a drug concentration of 800 µm, and the TAC and TACD groups received concentrations of 800 and 800 nm, respectively. An absorbable gelatin sponge (Gelatamp, COLTENE, Switzerland) was placed on each wound, and 10 µL of drug solution was administered each time. The wounds were further covered with Tegaderm (3M) and CobanTM (3M) bandages to reduce moisture loss and prevent animals from scratching. Images of the wounds were captured by camera (Canon 5D Mark IV, Japan) at fixed angles and focal lengths on days 0, 3, 6, 9, 12, and 15 and analyzed using Image J software. Samples of the tissues were collected and immediately fixed in 10% neutral‐buffered formalin on day 15. For further analysis, the samples were embedded in paraffin, sliced, and stained using standard histological protocols, including H&E, Masson, and immunofluorescence staining.

### Hematoxylin and Eosin Stain

Briefly, the tissue slices were routinely deparaffinized in water (placed in a 65 °C baking machine for 1 h, then immersed in xylene for 15 min, followed by fresh xylene deparaffinization for 15 min, and finally placed in gradient alcohol for tissue hydration and slicing, rinsed with water for 1 min). Then, they were stained with hematoxylin for 2 min and rinsed with water. After differentiation with hydrochloric acid alcohol for a few seconds and blueing with ammonia water for a few seconds, the sections were rinsed with water, then stained with eosin for 2 min, dehydrated in gradient alcohol, made transparent with xylene, and sealed with neutral gum.

### Biosecurity Assessment

Eight‐week‐old C57BL/6 mice were used for biosecurity assessment, and drugs were administered through the tail vein for seven consecutive days: 100 µL of sterile saline as the control group, 100 µL of 500 µM DMOG solution, 100 µL of 800 nM TAC, and 100 µL of 800 nM TACD. After 7 days, the mice were euthanized for tissue histopathological evaluation. Organs, including the heart, liver, spleen, lungs, and kidneys, were fixed in 4% paraformaldehyde, embedded in paraffin, and sectioned for H&E staining.

### Masson‐Trichrome Staining

Sections were routinely dewaxed in water and stained with premade Weigert's iron hematoxylin staining solution for 10 min. After thorough rinsing in water, sections were differentiated in hydrochloric acid alcohol for several seconds. After counterstaining with Masson's blue solution for 5 min, the sections were washed in water. Subsequently, they were stained with Light Green SF Yellowish staining solution for 10 min, washed in weak acid working solution for 1 min, and washed in 1% phosphomolybdic acid solution for 2 min. After staining in Aniline Blue staining solution for 1–2 min, the sections were washed in weak acid working solution for 1 min. Finally, the sections were dehydrated in ethanol, cleared in xylene, and mounted with neutral gum.

### Immunofluorescence (Histology)

The slices were routinely dewaxed into water. The slices were then soaked in antigen repair solution and placed in a 95 °C water bath for 20 min. Afterward, they were placed at room temperature and cooled to room temperature before being rinsed with PBS. To permeabilize, 0.5% Triton X‐100 was added dropwise for 15 min. After rinsing with PBS, 5% BSA was added and incubated at room temperature for 20 min for blocking. The primary antibody dilution solution was added and incubated overnight at 4 °C. The primary antibodies used were HIF‐1 alpha (ab308433, Abcam); VEGFA (ab52917, Abcam); VEGF Receptor 2 (ab2349, Abcam); CD31 (ab222783, Abcam); COL1‐A1 (ab21286, Abcam); alpha‐SMA (ab7817, Abcam). On the second day, after rinsing with PBS, the fluorescent secondary antibody and DAPI dilution solution were added and incubated at room temperature in the dark for 2 h. The slides were then rinsed with PBS in the dark and sealed.

### Statistical Analysis

Data were presented as the mean ± standard deviation (SD) (n ≥ 3). Differences between groups were assessed using a *t*‐test. GraphPad Prism 7.0 and ImageJ were used for statistics and analysis. Statistical analysis: *p*‐values < 0.05 were statistically significant. ^#/ */ @/ &/^ *p* < 0.05, ^##/ !!/ **/ @@/ &&/^ *p* < 0.01, ^###/ ***/ @@@/ &&&/^ *p* < 0.001.

## Conflict of Interest

The authors declare no conflict of interest.

## Author Contributions

Y.G. and Q.W. contributed equally to this work. X.C. and Y.L. supervised and conceived of the study. Y.G. and Q.W. designed and completed the experiments. Y.G. and Q.W. collected and analyzed the data. Y.G. wrote the manuscript. Y.Y., X.Q., J.S., and W.C. provided help during data collection and analysis.

## Supporting information

Supporting Information

## Data Availability

The data that support the findings of this study are available from the corresponding author upon reasonable request.
