# Supplementary material for: Framework Nucleic Acids‐Based VEGF Signaling Activating System for Angiogenesis: A Dual Stimulation Strategy
Source: Adv Sci (Weinh). 2024 Mar 9;11(21):2308701. doi: 10.1002/advs.202308701 (PMC11151028; doi:10.1002/advs.202308701)
Supplement: Supplementary file 1 — Supporting Information [file ADVS-11-2308701-s001.pdf]

ADVANCED SCIENCE
Open Access

Supporting Information

for *Adv. Sci.*, DOI 10.1002/advs.202308701

Framework Nucleic Acids-Based VEGF Signaling Activating System for Angiogenesis: A Dual Stimulation Strategy

*Yichen Ge, Qingxuan Wang, Yangxue Yao, Qin Xin, Jiafei Sun, Wen Chen, Yunfeng Lin and Xiaoxiao Cai**

Framework Nucleic Acids-Based VEGF Signalling Activating System for Angiogenesis: A Dual Stimulation Strategy

*Yichen Ge[1], Qingxuan Wang[1], Yangxue Yao[1], Qin Xin[1], Jiafei Sun[1], Wen Chen[1], Yunfeng Lin[1,2], Xiaoxiao Cai[1,2]**

1. State Key Laboratory of Oral Diseases, National Center for Stomatology, National Clinical Research Center for Oral Diseases, West China Hospital of Stomatology, Sichuan University, Chengdu, Sichuan 610041, China

2. Sichuan Provincial Engineering Research Center of Oral Biomaterials, Chengdu, Sichuan 610041, China

5’ 3’

S1: CATGCGAGGGTCCAATACCGACGATTACAGCTTGCTACACGCCTATCAGCTGGCCCCCCCC

Int Azide dT

S2: CCTATCAGCTGGCCCCCCCCCCGTGTAGCAAGCTGTAATCGACGGGAAGAGCATGCCCATCC

Int Azide dT

S3: ACGGTATTGGACCCTCGCATGCCTATCAGCTGGCCCCCCCCGGATGGGCATGCTCTTCCCG

Int Azide dT

S4-1/3: AGCTGATAGG GGGGGGGCC

S4-2/3: AGCTGATAGG GGGGGGGCC AGCTGATAGG GGGGGGGCC

S4: GGGGGGGCCAGCTGATAGGGGGGGGGCCAGCTGATAGGGGGGGGGCCAGCTGATAGG

Sapt: AGCTGATAGGATGGGTTGTAGGTCTAGGGGGGGCC

Figure S1. Sequences of the Oligonucleotides and the azide modification sites

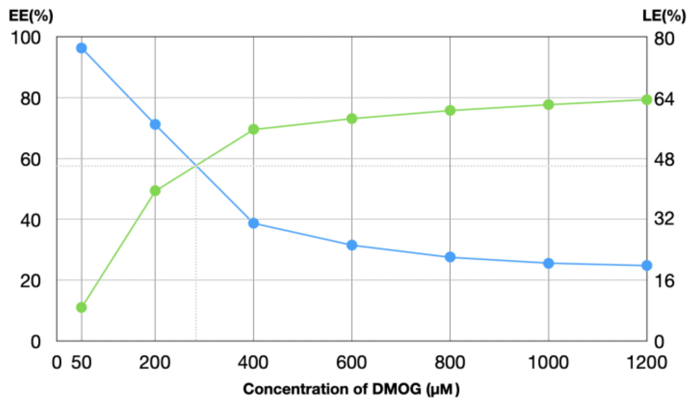

Figure S2. Calculation of encapsulation efficiency and loading efficiency

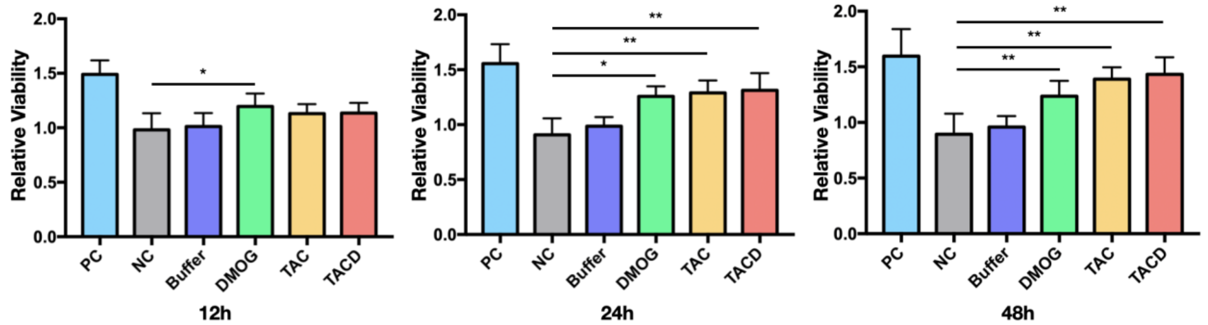

Figure S3. Cell viability of HUVECs after drug treatment for 12h, 24h and 48h. Data are presented as the mean ± standard deviation (SD) (n = 6) **P < 0.01, *P < 0.05 (Student's t-test).

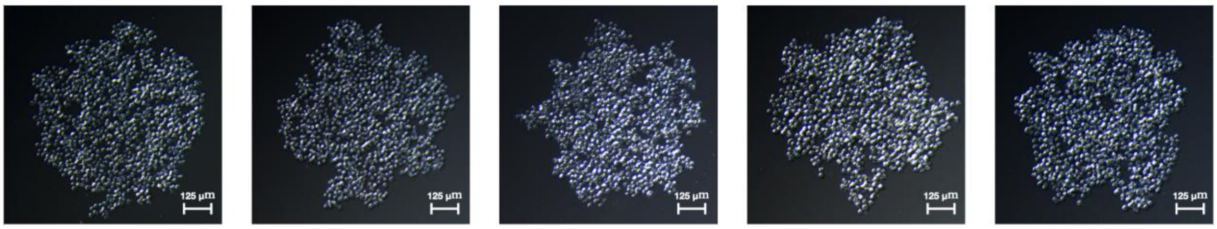

Figure S4. Cell aggregates induced in agarose gel presenting similar size.

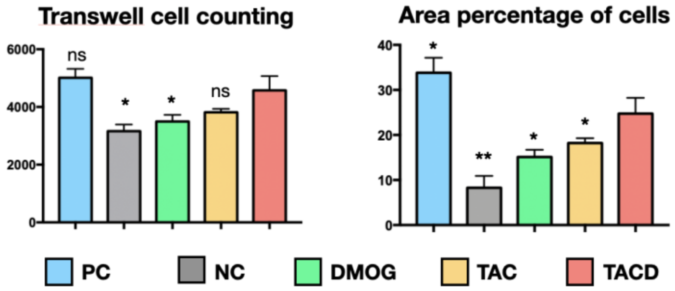

Figure S5. Quantitative analysis of crystal violet staining images. Data are presented as the mean ± standard deviation (SD) (n = 3). **P < 0.01, *P < 0.05 compared with TACD (Student's t-test).

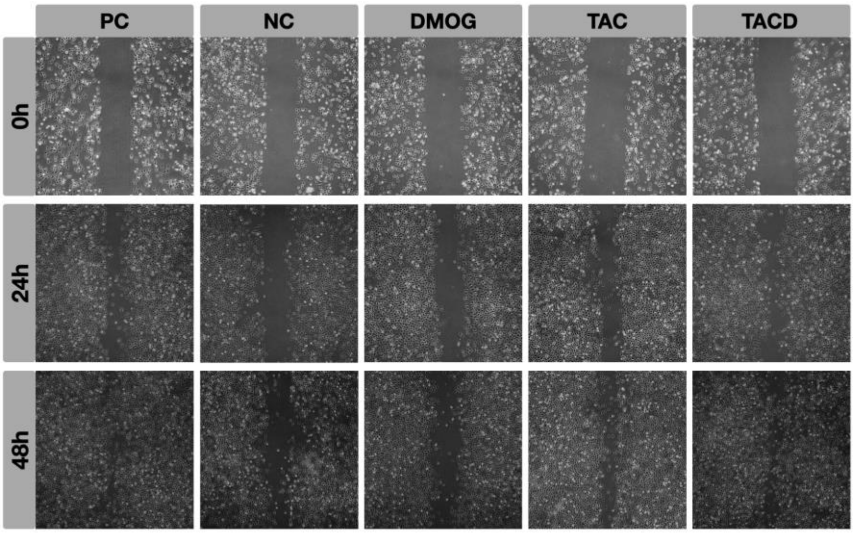

Figure S6. The images of scratch assay at 0h, 24h and 48h.

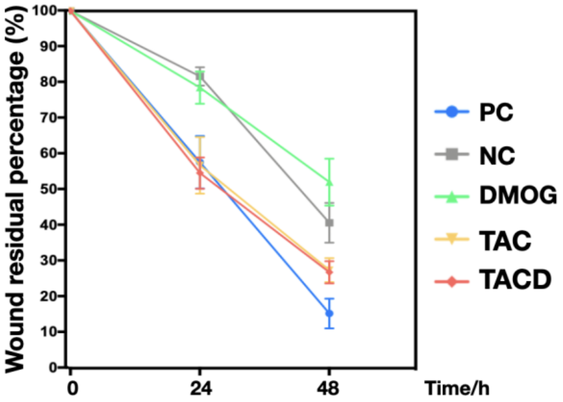

Figure S7. Quantitative analysis of wound residual percentage. Data are presented as the mean ± standard deviation (SD) (n = 3).

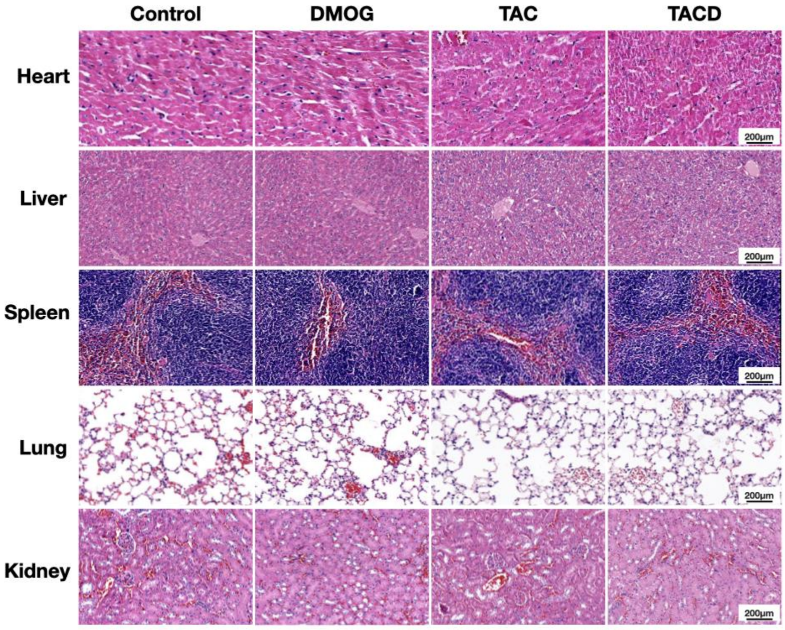

Figure S8. Representative images of histopathological sections of various organs after 7 days drug treatment.
